# Stereoscopy Amplifies Emotions Elicited by Facial Expressions

**DOI:** 10.1177/2041669515615071

**Published:** 2015-11-20

**Authors:** Jussi Hakala, Jari Kätsyri, Jukka Häkkinen

**Affiliations:** Department of Computer Science, Aalto University, Espoo, Finland; Institute of Behavioural Sciences, University of Helsinki, Finland

**Keywords:** Face perception, three-dimensional, mentalizing, theory of mind, self-relevance, presence

## Abstract

Mediated facial expressions do not elicit emotions as strongly as real-life facial expressions, possibly due to the low fidelity of pictorial presentations in typical mediation technologies. In the present study, we investigated the extent to which stereoscopy amplifies emotions elicited by images of neutral, angry, and happy facial expressions. The emotional self-reports of positive and negative valence (which were evaluated separately) and arousal of 40 participants were recorded. The magnitude of perceived depth in the stereoscopic images was manipulated by varying the camera base at 15, 40, 65, 90, and 115 mm. The analyses controlled for participants’ gender, gender match, emotional empathy, and trait alexithymia. The results indicated that stereoscopy significantly amplified the negative valence and arousal elicited by angry expressions at the most natural (65 mm) camera base, whereas stereoscopy amplified the positive valence elicited by happy expressions in both the narrowed and most natural (15–65 mm) base conditions. Overall, the results indicate that stereoscopy amplifies the emotions elicited by mediated emotional facial expressions when the depth geometry is close to natural. The findings highlight the sensitivity of the visual system to depth and its effect on emotions.

## Introduction

An observer is able to infer the mental state and intentions of a person based on facial expressions. For example, a smile might invite interaction, while a frown signals the opposite. Deciphering facial expressions is an aspect of mentalizing, or theory of mind, that is, the process of inferring the mental states of others. Emotional facial expressions have received substantial attention in a number of research fields due to their role in avoiding threat (e.g., Öhman & Mineka, 2001) and their importance in social interaction (e.g., Frith, 2009) and evoking empathy (e.g., Carr, Iacoboni, Dubeau, Mazziotta, & Lenzi, 2003). In nonmediated real-life situations, the evolutionary benefits of mentalizing are clear because the other person is able to physically interact with the observer.

In mediated communication, however, the other person’s mental state is less relevant to the observer’s immediate behavior. Although a picture of a face might evoke the same cognitive representation as a live face, the observer is simultaneously aware of the medium, for example, aware that the face is presented as a picture on a computer display. Pönkänen, Alhoniemi, Leppänen, and Hietanen (2011) and Pönkänen et al. (2008) demonstrated that the human brain processes live faces and pictures of faces differently even at early stages of processing. Their first study measured the face-sensitive event-related potential component N170 elicited by faces and dummy faces in live and picture conditions. Faces elicited stronger N170 responses than dummy faces but only in the live condition. Their second study compared direct and averted gaze in live and picture conditions. Again, the elicited event-related potential difference between the direct and averted gaze was significant only in the live condition. Furthermore, in both studies, the live condition elicited differences in emotional valence self-ratings that were absent in the picture condition.

Photographs of facial expressions have been used in human emotion research for over a century (Watson, 2004), and static two-dimensional images of faces remain the method of choice in many emotion studies (cf. Ekman & Friesen, 1976; Goeleven, De Raedt, Leyman, & Verschuere, 2008). During the latter part of the 20th century, researchers began to focus increasing attention on other perceptual cues provided by facial expressions. In particular, the role of motion in recognizing emotional facial expressions has recently become a major area of interest (e.g., Krumhuber, Kappas, & Manstead, 2013). Because motion is omnipresent in most live situations, a dynamic presentation of a facial expression replicates the live situation more closely than a static presentation. One study found that dynamic presentation enhanced the perception of intense angry and sad facial expressions (Harwood, Hall, & Shinkfield, 1999), but the intense happy expressions in the experiment were so easy to recognize that no differences between the conditions were found. Ambadar, Schooler, and Conn (2005) demonstrated that motion facilitates the perception of all facial expressions (including happy expressions) in pictures in which the expressions of emotion are subtle. The authors attributed the benefit of motion to sensitivity to change rather than sensitivity to the characteristic movements associated with individual emotional expressions. Motion also significantly enhances the recognition of subtle emotions in animated facial expressions (Kätsyri & Sams, 2008). Later, research revealed that in addition to change sensitivity, the specific dynamics associated with facial expressions (i.e., motion cues) facilitate the recognition of emotions in pictures of facial expressions (Bould, Morris, & Wink, 2008). Because the motion cues improved recognition accuracy only for subtle facial expressions, the authors concluded that intense facial expressions are recognizable regardless of other cues. However, intense dynamic facial expressions elicit higher experienced emotional arousal (Sato & Yoshikawa, 2007) and activity in brain regions associated with emotional processing (Arsalidou, Morris, & Taylor, 2011) than do static facial expressions.

Similar to motion, the stereoscopic presentation of faces might modulate the interpretation of facial expressions because stereoscopic vision supports more accurate perception of the three-dimensional structure of a face. In both stereoscopic imaging systems and live situations, the visual system fuses the images from the two retinae of the observer into a single three-dimensional cyclopean percept. The internal representations of faces include three-dimensional information (Jiang, Blanz, & O’Toole, 2009), which might explain why stereoscopy enhances facial recognition (Burke, Taubert, & Higman, 2007). Moreover, stereoscopy increases experienced presence when the depth magnitude is natural (IJsselsteijn, de Ridder, Hamberg, Bouwhuis, & Freeman, 1998; Takatalo, Kawai, Kaistinen, Nyman, & Häkkinen, 2011). The magnitude of depth in a stereoscopic image depends on the distance between the optical axes of the left and right cameras (i.e., the camera base). When the viewing distance and retinal size of the picture and the physical world scene are equal and the camera base is equal to the interpupillary distance of the observer, the capture and viewing geometries are identical. Increasing or decreasing the camera base results in the flattening or stretching of the image, respectively, along the depth axis. This distortion is typically characterized as the roundness factor (Devernay & Beardsley, 2010), that is, the ratio of the perceived depth from binocular disparity to actual object depth.

Surprisingly, the literature has neglected the role of stereoscopy in perceiving and responding to pictures of emotional facial expressions. In the present study, we investigate the connection between stereoscopy and the emotions elicited by emotional facial expressions. Our primary hypothesis is that stereoscopy amplifies the emotional response elicited by facial expressions due to the addition of three-dimensional cues and increased presence. In addition, we explore the effect of depth magnitude on the emotions elicited. Because the illusion of nonmediation is at a maximum at the natural depth magnitude, our secondary hypothesis is that the benefit of stereoscopy peaks at the natural camera base.

## Method

### Stimulus Acquisition

**Figure 1.**
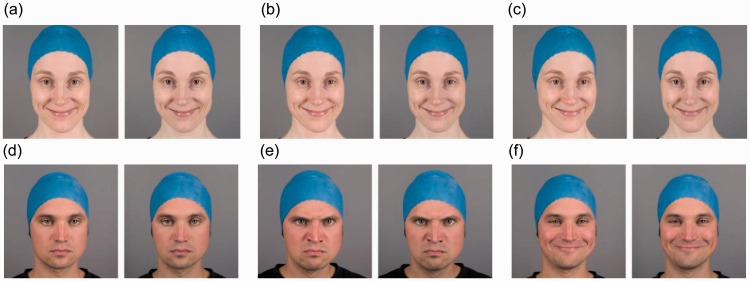
Sample stereoscopic stimuli arranged for parallel viewing. The top row exhibits a happy facial expression at the 15 mm (a), 65 mm (b), and 115 mm (c) camera bases. The bottom row exhibits neutral (d), angry (e), and happy (f) facial expressions at a 65 mm camera base.

Stereoscopic photographs of two females (F1, F2) and two males (M1, M2) professional actors served as experimental stimuli. Figure 1 presents examples of the stimuli. We used two canon 5D Mark II (Canon Inc., Tokyo, Japan) cameras with a 50-mm f/1.4 USM lens attached in a parallel configuration to a beam splitter stereo rig. The distance from the right camera focal point to the eyes of the model was 80 cm. The actors were instructed to look straight at the right side camera lens at all times. The right side camera was positioned directly in front of the actors’ face, while the left camera was positioned from 15 to 115 mm left of center at 25 mm intervals. Thus, the resulting camera bases were 15, 40, 65, 90, and 115 mm. The actors wore a rubber cap to cover their hair and other external facial features that might otherwise introduce variability into the stimuli and draw attention away from internal facial features (Gronenschild, Smeets, Vuurman, van Boxtel, & Jolles, 2009). In addition to neutral facial expressions, we photographed angry and happy facial expressions. We also attempted to standardize the facial expressions across different actors and different shots of the same actor. Recording sessions were supervised by one of the authors (JK), who is a certified facial action coding system (Ekman, Friesen, & Hager, 2002) coder. The target facial configurations were AU4 + 5 + 7 + 24 (for anger) and AU6 + 12 (for happiness). The photographs were cropped to square aspect ratio and shifted, so that the midpoint between the actors’ pupils in the left and right photographs was in the middle of the image. Thus, the eyes of the actors had zero stereoscopic disparity and were perceived at the display plane in the resulting stereoscopic image. The photographs were scaled to ensure that the distance between the pupils in the right side photograph on the display screen approximately matched the actors’ interpupillary distance.

### Stimuli and Apparatus

**Figure 2.**
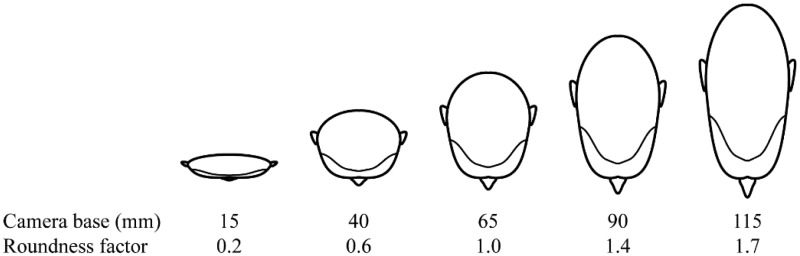
A simplified illustration of the effects of the camera base values used in the present study on the perceived depth cue from binocular disparity. The roundness factor, that is, the ratio of perceived depth from binocular disparity to the actual depth of the object, is calculated for an observer with a 65 mm interpupillary distance at 80 cm convergence distance. To observe the effect, see the 15, 65, and 115 mm stereoscopic sample stimuli presented in Figure 1.

The experiment was conducted in a laboratory setting with controlled lighting. The display area surrounding the stimulus photograph and the wall behind the display were gray and had a luminance of 15 cd/m^2^. The display device was a 24-inch autostereoscopic display SL2400 (Tridelity AG, St. Georgen, Germany) positioned 80 cm in front of the participants. Figure 2 illustrates the effect of camera base on the perceived stimuli at the 80 cm viewing distance. To restrict participants’ head movements and consequent image ghosting effects while viewing the autostereoscopic display, a chin rest was used throughout the experiment. Each participant was shown photographs of one male and one female actor. Participants were randomly assigned to one of the four different actor combinations (M1–F1, M1–F2, M2–F1, and M2–F2) such that an equal number of male and female participants saw each combination. The photographs were displayed under three stereo conditions: stereoscopic, monoscopic left, and monoscopic right. The three stereo conditions, five camera bases, three facial expressions, and two actors resulted in 90 trials per participant. Half of the participants viewed the original images, and the other half viewed images mirrored around the vertical axis. The participants recorded their responses on a tablet computer that was attached to the table in front of them.

### Participants

Twenty males and twenty females participated in the experiment; the mean age of participants was 28.1 years (*SD* = 9.5). The participants were of the same ethnic origin as the actors depicted in the stimuli. All participants exhibited normal or only slightly impaired visual acuity based on the Lea Numbers Test (Lea-Test Ltd, Helsinki, Finland). For four participants, visual acuity was below 20/20 (1.0 on the decimal scale) but above 20/40 (0.5). Near phoria was tested using the Maddox Wing test (Clement Clarke Ltd, London, UK); all participants measured less than 10 prism diopters horizontal and less than 2 prism diopters vertical phoria. All participants exhibited normal or only slightly impaired stereoacuity. Four participants failed the TNO stereo test (Laméris Ootech BV, Utrecht, The Netherlands) but performed well on the RANDOT stereo test (Stereo Optical Company Inc., Chicago, IL, USA). The mean interpupillary distance at the 80 cm viewing distance was 60.4 mm (*SD* = 2.7 mm). In addition to visual screening, participants completed the balanced emotional empathy scale (BEES; Mehrabian, 2000) and the 20-item Toronto alexithymia test (TAS-20; Bagby, Parker, & Taylor, 1994). The mean BEES score was 42.3 (*SD* = 26.5), and the mean TAS score was 45.3 (*SD* = 9.2). The present study adhered to the tenets of the World Medical Association Declaration of Helsinki and the ethical principles established by the Finnish Advisory Board on Research Integrity (http://www.tenk.fi/en/).

### Procedure

Participants evaluated photographs that were displayed on the screen. No explicit time limit was imposed on the evaluations. Participants used a stationary tablet computer that was positioned in front of them to record their responses to the 9-point self-assessment manikin (SAM) valence and arousal scales (Bradley & Lang, 1994; Lang, 1980). They were instructed to evaluate the emotional experience evoked by the stimuli. Although the original SAM scale implicitly posits that positive and negative valence activation is reciprocal by measuring valence on a single bipolar scale, research has found that a single scale is sometimes insufficient because ambivalent emotions include both positive and negative valence components (Cacioppo, Gardner, & Berntson, 1997, 1999). Thus, in contrast to the original SAM scales, we separated the valence scale into positive and negative scales. Whereas the original scale ranged from unhappy to happy with intervening neutral manikins, the positive and negative scales used in the present study ranged, respectively, from neutral to happy and from neutral to unhappy manikins. No verbal labels were attached to the scale values. Furthermore, the arousal and valence figures were presented with the scale values increasing from left to right to facilitate the use of the scales. Other variables that were not within the scope of the present study were also measured. Participants completed two practice trials prior to the 90 randomized experiment trials. The mean duration of experimental sessions was 41 min (*SD* = 9 min); the stereoscopic trials lasted longer (*M* = 28.6 s, *SD* = 11.3 s) than the monoscopic trials (*M* = 24.9 s, *SD* = 9.5 s).

### Analysis

The data were analyzed using R (R Core Team, 2015). To analyze the valence and arousal data, we used the lme4 R package (Bates, Maechler, Bolker, & Walker, 2015) implementation of linear mixed models (LMMs) with restricted maximum likelihood estimates. LMM analysis was used rather than more conventional methods such as analysis of variance because it enabled us to more appropriately specify error variance, and LMM analysis can be viewed as a generalization of the more restricted analysis of variance (Quené & Van Den Bergh, 2004). The degrees of freedom were Satterthwaite approximations. The contrast between the stereo and mono conditions was calculated as the difference between the mean of the left and right monoscopic conditions and the stereoscopic condition. Significance tests of the differences between contrasts were performed using Holm–Bonferroni (Holm, 1979) adjusted *p* values. The predictors used in all the models were stereoscopy, camera base, facial expression, participants’ gender, gender match (same/opposite), BEES score, and TAS score. The last four variables were used to control for potential confounds in the results because evaluations of emotional facial expressions have been found to be influenced by gender (Montagne, Kessels, Frigerio, De Haan, & Perrett, 2005), gender match (Vrana & Gross, 2004), empathy (Besel & Yuille, 2010), and alexithymia (Parker, Taylor, & Bagby, 1993). We also included interaction effects for all two-way interactions involving either stereoscopy or facial expression and all three-way interactions involving stereoscopy and facial expression. Random intercepts and facial expression slopes were defined for the individual actors to control for variability in individual actor expressions. In addition, random intercepts were specified for participants. The significance threshold was set to 5% in all analyses.

## Results

### Manipulation Check

**Figure 3.**
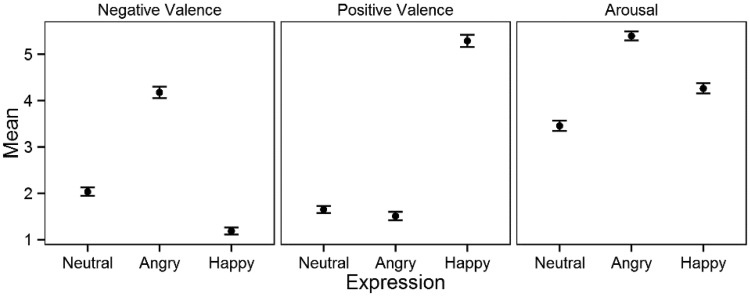
Marginal means of the three measured dimensions for the three facial expressions (95% CI).

To assess whether the facial expression stimuli elicited the expected emotional reactions, we analyzed the effects of facial expression type on negative valence, positive valence, and arousal ratings using LMM analyses. These manipulation checks were conducted for mean values that were pooled across monoscopic display conditions (i.e., left and right monoscopic images). Figure 3 presents comparisons of the marginal means of the emotional ratings of the facial expressions. Facial expression exerted a significant effect on negative valence, *F*(2, 32) = 54.9, *p* < .001, positive valence, *F*(2, 16) = 108.4, *p* < .001, and arousal, *F*(2, 114) = 7.83, *p* < .001. Contrasts between the facial expressions confirmed that the self-reported emotions were consistent with the presented facial expressions. As expected, angry expressions elicited higher negative valence than neutral and happy expressions, and happy expressions elicited significantly higher positive valence than neutral and angry expressions. Neutral expressions also elicited higher negative valence than happy expressions. Angry expressions elicited higher arousal than happy and neutral expressions, and happy expressions elicited higher arousal than neutral expressions. We defined valence ambiguity, on a scale from 1 to 9, as the minimum of positive and negative valence ratings (Kaplan, 1972; see also Viinikainen, Kätsyri, & Sams, 2012). None of the stimuli elicited highly ambiguous emotions; the maximum ambiguity score was 2.15.

### Negative Valence

**Figure 4.**
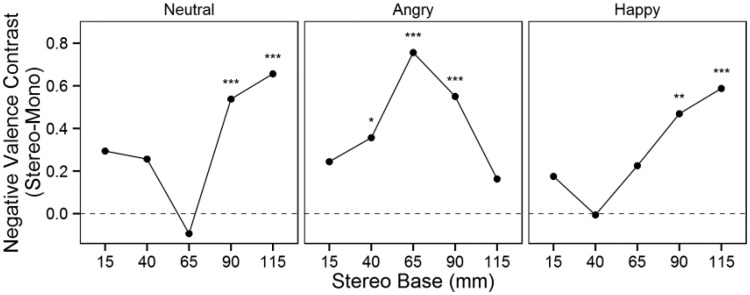
Negative valence contrasts between stereoscopic and monoscopic conditions. *p < .05. **p < .01. ***p < .001, two-tailed.

The LMM analysis for negative valence revealed a significant three-way interaction between stereoscopy, camera base, and facial expression, *F*(16, 3474) = 1.80, *p* = .030. Figure 4 illustrates the results of planned contrasts between the stereoscopic and monoscopic conditions (averaged across the left and right image conditions) for different facial expressions at different camera bases. The stereoscopic neutral and happy expressions elicited significantly higher negative valence at the 90 mm and 115 mm camera bases compared with the monoscopic conditions. For the angry expression, stereoscopy significantly increased the negative valence at the 40, 65, and 90 mm camera bases but not at the extreme 15 mm and 115 mm camera bases.

We used planned comparisons to assess the extent to which differences in the camera bases influenced the effect of stereoscopic compared with monoscopic presentation. All comparisons were made to the 65 mm camera base, which was expected to provide the most natural depth percept because it was closest to the mean participant interpupillary distance. For the neutral expression, stereoscopy significantly increased the negative valence of all the other camera bases compared with the 65 mm camera base (Table 1). For the angry facial expression, stereoscopy increased the negative valence significantly more at the 65 mm camera base compared with the contrasts at the 15, 40, and 115 mm camera bases. The 65 mm contrast did not differ significantly from the contrasts at other camera bases for the happy expression, and stereoscopy exerted the smallest effect on negative valence at the 40 mm camera base.

**Table 1.** Contrast Comparisons for Negative Valence.

Expression	Contrast comparison	Estimate	*SE*	*z* ratio	Adj. *p*	Sig.
Neutral	15 vs. 65	0.387	0.160	2.417	.031	*
	40 vs. 65	0.350	0.160	2.183	.029	*
	90 vs. 65	0.631	0.160	3.937	<.001	***
	115 vs. 65	0.750	0.160	4.678	<.001	***
Angry	15 vs. 65	−0.512	0.160	−3.197	.004	**
	40 vs. 65	−0.400	0.160	−2.495	.025	*
	90 vs. 65	−0.206	0.160	−1.286	.198	
	115 vs. 65	−0.594	0.160	−3.704	<.001	***
Happy	15 vs. 65	−0.050	0.160	−0.312	.755	
	40 vs. 65	−0.231	0.160	−1.442	.298	
	90 vs. 65	0.244	0.160	1.520	.385	
	115 vs. 65	0.363	0.160	2.261	.095	

*Note.* The contrasts for each camera base were compared with the reference level for each facial expression; *p* values were Holm–Bonferroni adjusted to control for multiple comparisons for each expression.

* *p* < .05. **p < .01. ***p < .001, two-tailed.

Participant gender exhibited a significant interaction with facial expression, *F*(2, 3377) = 8.5, *p* < .001. Neutral and angry expressions elicited significantly higher negative valences in female participants compared with male participants. Gender match also exhibited a significant main effect, *F*(1, 3474) = 19.6, *p* < .001. Actors of the opposite gender elicited lower negative valences than actors of the same gender. Furthermore, facial expression exhibited significant two-way interactions with the BEES score, *F*(2, 2848) = 12.2, *p* < .001, and the TAS score, *F*(2, 3476) = 10.1, *p* < .001. Higher BEES scores predicted higher negative valences for neutral facial expressions, and higher TAS scores predicted lower negative valences for angry and neutral expressions. Although the main effects of camera base and facial expression were also significant, we interpret the effects of those predictors only within the significant interactions in which they were involved (see above).

### Positive Valence

**Figure 5.**
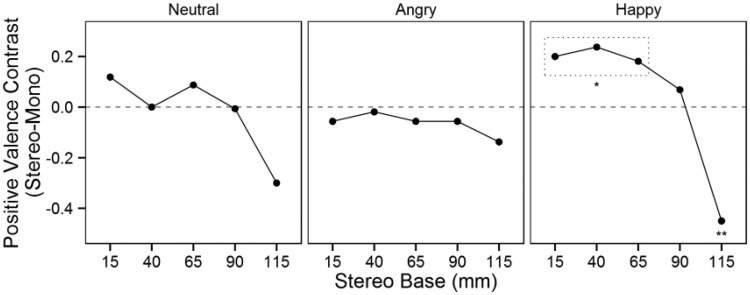
Positive valence contrasts between the stereoscopic and monoscopic conditions. The dotted rectangle highlights the three camera bases for the happy facial expression condition that, in combination, differed significantly between the monoscopic and stereoscopic conditions. *p < .05. **p < .01, two-tailed.

For positive valence, the three-way interaction between stereoscopy, camera base, and facial expression was not significant, *F*(4, 3474) = 1.1, *p* = .377. However, significant two-way interactions were found for stereoscopy and camera base, *F*(8, 3473) = 2.6, *p* = .009, and for camera base and facial expression, *F*(8, 3474) = 2.0, *p* = .045. Because the interaction between camera base and facial expression was inexplicable in itself and three-way interaction contrasts were planned, we analyzed the three-way interaction rather than the two-way interactions. Figure 5 presents the contrasts between the stereoscopic and monoscopic conditions. The neutral and angry expressions did not differ significantly between the stereoscopic and monoscopic conditions, but the contrast for the neutral facial expressions approached significance at the 115 mm camera base, *z* = −1.87, *p* = .062. For the happy expression, the 115 mm stereoscopic condition elicited significantly lower positive valence than the monoscopic condition, *z* = −2.8, *p* = .005. The contrasts for the 15, 40, and 65 mm camera bases in the happy expression also warranted further inspection. A post hoc contrast of the happy facial expression revealed that the combined positive valence of the three smallest camera bases (15, 40, 65 mm) was significantly higher in the stereoscopic condition than in the monoscopic condition, *z* = 2.22, *p* = .026. For the happy facial expression, stereoscopy increased the positive valence most at the 40 mm camera base. The planned comparison of contrasts using the 65 mm contrast as a reference yielded only a single significant difference following Holm–Bonferroni adjustment (Table 2); compared with the monoscopic condition, stereoscopy induced a significantly higher increase in positive valence for the happy expression at the 65 mm camera base than at the 115 mm camera base.

**Table 2.** Contrast Comparisons for Positive Valence.

Expression	Contrast comparison	Estimate	*SE*	*z* ratio	Adj. *p*	Sig.
Neutral	15 vs. 65	0.031	0.161	0.194	.846	
	40 vs. 65	−0.088	0.161	−0.544	1	
	90 vs. 65	−0.094	0.161	−0.583	1	
	115 vs. 65	−0.387	0.161	−2.411	.064	
Angry	15 vs. 65	0.000	0.161	0.000	1	
	40 vs. 65	0.038	0.161	0.233	1	
	90 vs. 65	0.000	0.161	0.000	1	
	115 vs. 65	−0.081	0.161	−0.506	1	
Happy	15 vs. 65	0.019	0.161	0.117	.907	
	40 vs. 65	0.056	0.161	0.350	1	
	90 vs. 65	−0.113	0.161	−0.700	1	
	115 vs. 65	−0.631	0.161	−3.928	.000	***

*Note.* The contrasts for each camera base were compared with the reference level for each facial expression; *p* values were Holm–Bonferroni adjusted to account for multiple comparisons within each expression.

*** *p* < .001, two-tailed.

There were significant interactions between facial expression and BEES score, *F*(2, 3438) = 11.2, *p* < .001, and between facial expression and TAS score, *F*(2, 3475) = 32.2, *p* < .001. Higher BEES scores predicted higher positive valence ratings for neutral expressions, whereas higher TAS scores predicted lower positive valence ratings for happy expressions. The interaction between gender match and facial expression was significant, *F*(2, 3474) = 5.6, *p* = .004. Angry expressions of the opposite gender elicited significantly higher positive valence compared with those of the same gender. The interaction between participants’ gender and facial expression was also significant, *F*(2, 3475) = 39.8, *p* < .001. The happy facial expression condition elicited significantly higher positive valence in female participants compared with male participants.

### Arousal

**Figure 6.**
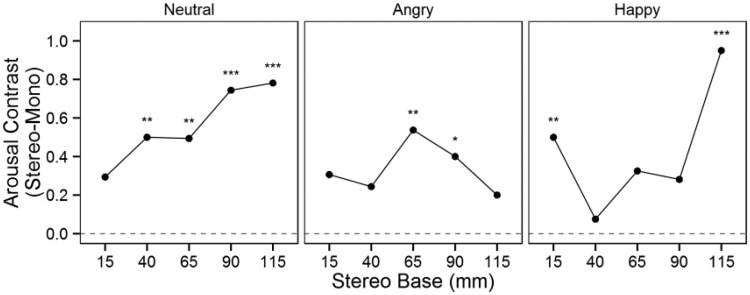
Arousal contrasts between the stereoscopic and monoscopic conditions. *p < .05. **p < .01. ***p < .001, two-tailed.

Figure 6 presents comparisons of the arousal contrasts between the stereoscopic and monoscopic conditions. Stereoscopy significantly increased arousal to the neutral facial expression for all but the smallest camera base. For angry facial expressions, stereoscopy increased arousal at the 65 mm and 90 mm camera bases. For happy expressions, the extreme camera bases (15 mm and 115 mm) in the stereoscopic condition elicited significantly higher arousal compared with the monoscopic condition. Comparison of the contrasts with the 65 mm reference contrast revealed no significant differences apart from the 115 mm camera base contrast for the happy facial expression condition (Table 3). However, a post hoc comparison of the 40 mm contrast to the other contrasts for the happy facial expression revealed significant differences between the 15 mm and 40 mm conditions, *z* = 2.49, *p* = .038, and between the 40 mm and 115 mm conditions, *z* = 5.13, *p* < .001. There was a significant interaction between facial expression and BEES score, *F*(2, 3285) = 10.21, *p* < .001. Higher BEES scores predicted lower arousal ratings for happy facial expressions. There was also a significant interaction between facial expression and TAS score, *F*(2, 3477) = 6.16, *p* = .002. Higher TAS scores predicted lower arousal ratings for neutral expressions and higher arousal ratings for happy facial expressions.

**Table 3.** Contrast Comparisons for Arousal.

Expression	Contrast comparison	Estimate	*SE*	*z* ratio	Adj. *p*	Sig.
Neutral	15 vs. 65	−0.200	0.171	−1.172	.482	
	40 vs. 65	0.006	0.171	0.037	.971	
	90 vs. 65	0.250	0.171	1.465	.429	
	115 vs. 65	0.288	0.171	1.685	.368	
Angry	15 vs. 65	−0.231	0.171	−1.355	.351	
	40 vs. 65	−0.294	0.171	−1.721	.256	
	90 vs. 65	−0.138	0.171	−0.806	.420	
	115 vs. 65	−0.337	0.171	−1.978	.192	
Happy	15 vs. 65	0.175	0.171	1.026	.610	
	40 vs. 65	−0.250	0.171	−1.465	.429	
	90 vs. 65	−0.044	0.171	−0.256	.798	
	115 vs. 65	0.625	0.171	3.663	.001	**

*Note.* The contrasts for each camera base were compared with the reference level for each facial expression; *p* values were Holm–Bonferroni adjusted to account for multiple comparisons within each expression.

** *p* < .01, two-tailed.

## Discussion

We tested the hypotheses that stereoscopy enhances the emotional responses elicited by facial expressions, and that natural depth maximizes these effects. Stereoscopy significantly increased the negative valence elicited by angry facial expressions, and the increase was highest for photographs taken at the 65 mm camera base. Similarly, stereoscopy increased the arousal elicited by angry facial expressions most at the 65 mm camera base. The results were less distinct for happy facial expressions. Stereoscopy subtly increased the positive valence of happy expressions in photographs taken at the 15, 40, and 65 mm camera bases compared with the monoscopic photographs. The combined contrast of the three camera bases was statistically significant between the stereoscopic and monoscopic conditions, but the individual contrasts were nonsignificant. In contrast to angry expressions, the arousal elicited by happy expressions was amplified by stereoscopy only at the extreme 15 and 115 mm camera bases.

Stereoscopy might enhance the emotional response of observers through two mechanisms. First, similar to motion, stereoscopy provides additional visual cues for the recognition of facial expressions. Earlier research has demonstrated that face recognition benefits from stereoscopic presentation (Burke et al., 2007), possibly due to three-dimensional information in internal representations (Jiang et al., 2009); this might also hold for facial expressions. The effect of stereoscopy in our results was clearest for angry facial expressions, which might indicate that the stereoscopic cues enhanced recognition of angry expressions, while happy expressions were relatively easier to identify without the additional cues provided by stereoscopy. This interpretation is supported by previous findings that demonstrated that happy facial expressions are unambiguously recognized, whereas angry facial expressions are more often confused with other negative emotions (e.g., Palermo & Coltheart, 2004). Our results further indicate that experienced valence and arousal are highly sensitive to the magnitude of depth; the benefit of stereoscopy reached a maximum at the natural depth magnitude. These findings suggest the operation of a second mechanism, which is the increased illusion of nonmediation, that is, presence. IJsselsteijn et al. (1998) found that stereoscopy increases presence if the observer perceives the depth as natural. In the case of stereoscopic facial expressions, both the physical and social components of presence (IJsselsteijn, de Ridder, Freeman, & Avons, 2000) are relevant. The appraisal of self-relevance is another process that modulates the perception of emotional facial expressions (N’Diaye, Sander, & Vuilleumier, 2009). While social presence and self-relevance could be addressed separately (e.g., Blascovich et al., 2002), we propose that social presence increases self-relevance. As the physical and social presence elicited by a facial expression presented on a display screen increases, effectively transforming a picture into something more real, the stimulus becomes more self-relevant to the observer. Self-relevance might also explain the significantly stronger amplification of the valence elicited by stereoscopic angry facial expressions compared with happy facial expressions because angry expressions are more relevant to the immediate behavior of the observer than are happy expressions (Cacioppo et al., 1999).

The stereoscopic characteristics of the media present a challenge when assessing the effects of stereoscopy on emotions or presence. First, several artefacts (Boev, Hollosi, Gotchev, & Egiazarian, 2009) appear only in the stereoscopic rendition of the stimuli. These artefacts might be due to content creation, processing, or presentation. Second, the combination of the capture and viewing geometries affects the resulting depth percept. Although the roundness factor (Devernay & Beardsley, 2010) provides an estimate of the distortion due to binocular disparity, the visual system employs other depth cues, such as size, occlusion, and accommodation, that interact with each other. Individuals also vary in their interpupillary distance, which creates variability in the perceived depth produced by binocular disparity. Furthermore, the capture and viewing geometries also affect the fusibility of the image and possible eyestrain and discomfort (Lambooij, IJsselsteijn, Fortuin, & Heynderickx, 2009). The negative effects of media characteristics on presence are also included in a frequently used sense-of-presence questionnaire (Lessiter, Freeman, Davidoff, & Keogh, 2001) because they have been found to significantly decrease the sense of presence.

In interpreting the results of the present study, it is important to note that in addition to the fundamental difference between the stereoscopic and monoscopic conditions, the negative media characteristics identified above might contribute to the emotions elicited by stereoscopic stimuli. Research has demonstrated that increasing binocular disparity increases the self-reported arousal elicited by stereoscopic images (Kawai et al., 2014), possibly due to negative media characteristics. Large camera bases can produce eye discomfort, which might explain the increased negative valence elicited by the neutral and happy expressions at the high camera base levels found in the present study. Furthermore, crosstalk between images intended for the left and right eyes (Woods, 2012) becomes more visible with larger disparities. For the angry expressions, stereoscopy amplified arousal most at the midrange camera bases, which provided the most natural depth percept. The arousal results for the happy expressions were unexpected and warrant further investigation. Stereoscopy amplified the arousal elicited by happy expressions only at the extreme 15 and 115 mm camera bases, which produced the most unnatural depth percepts. The happy expression result might be explained by self-relevance. Because the happy expressions elicited positive valence, the participants might have felt annoyed by the depth distortion because it reduced the presence elicited by the stimulus and, consequently, reduced self-relevance. Another explanation for the increased arousal elicited by the small camera base is that there might not have been enough three-dimensionality: Users expect stereoscopic images to be fully three-dimensional (Hakala et al., 2014). The negative valence results for the neutral facial expressions are also noteworthy because they indicate that the natural 65 mm camera base elicited significantly less negative valence than the smaller or greater camera bases. This finding is a prime example of the negative effect of unnatural depth on elicited emotional valence. The effect was reversed for the angry expression: The natural 65 mm camera base elicited the highest amplification of negative valence. If the negative valence amplification were due exclusively to the adverse reaction to the negative media characteristics, the negative valence contrast would have increased monotonically with the camera base as the artefacts and distortions became more noticeable. However, our results show that the negative valence contrast curves resemble U or inverted U-shaped curves (Figure 4), indicating the special significance of the midrange natural camera bases.

In the present study, participants’ gender and gender match as well as alexithymia and emotional empathy traits were included as potential confounding factors. Our results indicated that for female participants, angry and neutral expressions elicited stronger negative valence, and happy expressions elicited stronger positive valence. Because both angry and neutral expressions elicited negative emotional reactions (Figure 3), these results can be considered as evidence of higher emotional sensitivity in female participants. This interpretation is confirmed by earlier findings. Compared with males, females have been found to be more accurate in recognizing angry and happy facial expressions (Hall & Matsumoto, 2004), mimic facial expressions more actively (Dimberg & Lundquist, 1990), and report higher positive valence for happy facial expressions and higher negative valence for sad expressions (Wild, Erb, & Bartels, 2001). The gender match results indicate that the expressions of the opposite gender elicit emotions that are more favorable (i.e., less negative). Consistent with these results, previous research has found that pictures of females elicit more positive valence in males than pictures of males (Vrana & Gross, 2004). However, because the stimuli in the present experiment depicted the posed emotional expressions of only two females and two males, gender-specific results must be interpreted with caution. The validity of the present emotional empathy and alexithymia-related results is also limited because these traits were measured in the randomly selected participant sample rather than employed as inclusion criteria. Nevertheless, most of the study results are consistent with previous findings. For example, alexithymia has been associated with difficulties in recognizing both one’s own and others’ emotions (Bagby et al., 1994), which is consistent with the present finding that angry and neutral (i.e., slightly negative) faces elicited fewer negative experiences and happy faces elicited fewer positive experiences in more alexithymic participants. It should be noted that gender, alexithymia, and empathy did not predict any of the responses elicited by stereoscopic displays.

Further studies are needed to confirm the mechanisms through which stereoscopy amplifies emotions. Including a live condition in the experimental setup in future studies would provide a baseline against which the stereoscopic and monoscopic conditions could be compared. Additionally, the combined effect of stereoscopy and dynamics is the next logical research focus in the endeavor to understand the gap between a picture and the reality that it represents.

## Conclusions

The present article presents evidence that stereoscopy amplifies self-reported emotions, but only when depth is natural. Stereoscopy increased the negative valence elicited by angry facial expressions and the positive valence elicited by happy facial expressions. Moreover, for angry facial expressions, the amplification in arousal caused by stereoscopy reached a maximum in the natural depth condition. We propose that stereoscopy amplifies emotional responses through two mechanisms. First, the additional visual cues provided by stereoscopy enhance perception of the facial expression. Second, stereoscopy increases the illusion of nonmediation and thus renders facial expression stimuli more self-relevant to the observer.

Our findings have implications for fields involving pictorial facial expressions, such as human–computer interaction, nonverbal communication, emotion psychology, and emotion neuroscience. The currently ongoing proliferation of head-mounted virtual reality displays appears to have generated a new boom in stereoscopic displays. Our findings should thus contribute to maximizing the emotional impact of stereoscopic content and increase popular acceptance of the new technology. Furthermore, the study findings provide an opportunity to revisit investigations of emotional facial expressions that were performed using pictorial stimuli.
